# Virtual Interactomics of Proteins from Biochemical Standpoint

**DOI:** 10.1155/2012/976385

**Published:** 2012-08-08

**Authors:** Jaroslav Kubrycht, Karel Sigler, Pavel Souček

**Affiliations:** ^1^Department of Physiology, Second Medical School, Charles University, 150 00 Prague, Czech Republic; ^2^Laboratory of Cell Biology, Institute of Microbiology, Academy of Sciences of the Czech Republic, 142 20 Prague, Czech Republic; ^3^Toxicogenomics Unit, National Institute of Public Health, 100 42 Prague, Czech Republic

## Abstract

Virtual interactomics represents a rapidly developing scientific area on the boundary line of bioinformatics and interactomics. Protein-related virtual interactomics then comprises instrumental tools for prediction, simulation, and networking of the majority of interactions important for structural and individual reproduction, differentiation, recognition, signaling, regulation, and metabolic pathways of cells and organisms. Here, we describe the main areas of virtual protein interactomics, that is, structurally based comparative analysis and prediction of functionally important interacting sites, mimotope-assisted and combined epitope prediction, molecular (protein) docking studies, and investigation of protein interaction networks. Detailed information about some interesting methodological approaches and online accessible programs or databases is displayed in our tables. Considerable part of the text deals with the searches for common conserved or functionally convergent protein regions and subgraphs of conserved interaction networks, new outstanding trends and clinically interesting results. In agreement with the presented data and relationships, virtual interactomic tools improve our scientific knowledge, help us to formulate working hypotheses, and they frequently also mediate variously important *in silico* simulations.

## 1. General Remarks

Many important findings in pharmacology, cell biology, and pathobiology have been achieved with the aid of virtual interactomics including computer-aided structural analysis, prediction and *in silico* simulation of interacting sites, protein complexes, and interaction networks. Virtual interactomics has been developed in the last thirty years, and it is in fact based on gradual bioinformatic processing of experimental data. These data were usually obtained from individual studies of interactions, and various large-scale experimental methods such as the two-hybrid system, phage display library studies reverse interactomics, SPOT arrays or microarray studies, and extended sequence studies [1–7]. 

In addition to sequence data, three-dimensional (3D) structures are ever more frequently required for interactomic predictions. X-ray crystallography or nuclear magnetic resonance studies represent the most frequent sources of 3D structures, whereas combination of electron microscopy of molecular complexes with X-ray crystallography turns out to be interesting for the same purpose [8–11]. Alternatively, sophisticated 3D structure simulations such as homology modeling or combination of cryoelectron microscopy densities, and molecular dynamics appear to be also useful for approximating conventional 3D input at least in some cases [12–14]. In addition to 3D shape, solvent and surface accessibilities (or more likely actual dynamic accessibility following from accompanying interacting structures or proteolysis; cf. [15, 16]) were considered to be important criteria for reevaluation of possible interaction sites. Many experimentally investigated and predicted structural relationships were also stored in interactomic databases to be selectively found, processed and compared. Moreover, some interaction data differently stored in multiple databases have been searched with the aid of special data mining servers such as Dasmiweb and PINA v2.0 ([17, 18]; see also Table 1).

Virtual interactomic studies are mostly realized via computer programs including numerous online accessible tools. Three types of computer data processing are important for online interactomic predictions on molecular level, that is, (i) structure comparisons, (ii) molecular docking studies, and (iii) reevaluation of current database data, and accessible or proposed protein interaction networks. Similarly, three types of interacting structures can be distinguished with respect to their different molecular origin. This concerns (i) conserved structures, (ii) randomly/quasi-randomly *in vitro* generated or rapidly evolved structures (e.g., mimotopes and disordered regions), and (iii) binding sites of antigen receptors expressed in specific immune cell clones, which are usually developed during the regular recombination process, and later also at the time of immune response.

In contrast to reactomics, interactomics described here deals exclusively with interactions, and thus concerns only interactions of enzyme active sites but not their subsequent reaction mechanism. Consequently, modeling of enzyme reactions exceeds the topic of this review. In addition, since we dealt here with protein interactomics, this paper also does not contain information about interactions of DNA with nonpeptide ligands.

## 2. Structural Similarities of Interacting Sites 

By using various structurally based programs, many protein interactions can be predicted based on the occurrence of phylogenetically conserved or convergently developed functionally important common structural features (motifs, sequence patterns, consensi, constructs of conserved domain sequences, supersecondary structures, supersecondary motifs, 3D-arranged structural patterns and pockets). However, diversification within protein families, and superfamilies causes losses of interacting structures or disables their accessibility. On the other hand, new interactive pairs frequently appear in cases of disordered protein regions, that is, peptide segments naturally occurring in multiple conformation variants [19–21]. The attendant stability problems, as well as some additional problems with molecular analysis, were diminished by the development of databases enabling reevaluation of selected structural relationships. The databases inform us about similar or common structural features, frequent locations of binding sites in related domains, solvent accessibility, and location of investigated segments in 3D structures of proteins (see Table 1).

During the last twenty years, conventional sequence-based search for conserved structures frequently combined different evaluations of sequence similarities. The corresponding protocols usually combined double sequence, and multiple sequence comparisons like BLASTP, PSI-BLAST, RPS-BLAST, Clustal W and MUSCLE [22, 23]. In addition, highly selective PHI-BLAST or PSI-BLAST searches with specifically restricted representative query sequences such as consensi (and also sequence patterns) made it possible to locate the corresponding potentially interacting sites in extended sequence sets [23–26]. Except for current conserved sequences, extremely variable but still defined structures such as heptad repeats were investigated for purposes of molecular topology [27, 28]. These repeats can be written as an alphabet with generalized characters representing aa groups instead of individual aa (hydrophobic, charged, polar, etc.). Together with usual (motif-related sequence block derived) patterns an important part of heptad repeats can be searched in database sequences and partially reevaluated by means of PROSITE programs [29–32]. Apart from regions with highly conserved sequences, additional conserved structures were found when combining evaluation of primary and secondary structures [33], PSI-BLAST and secondary structure [34], or when using fold recognition [34, 35]. The compared sequence queries were moreover evaluated on the sophisticated widely used FFAS03 server (about 250 references) providing the third generation of the profile-profile alignment and fold-recognition algorithm mediated by program FFAS (fold, and function assignment system; [36, 37]; Table 1). The sensitivity FFAS-related profile-profile comparison is now widely recognized and many Web servers implementing such algorithms are available, for example, HHPRED, COMPASS, COMA, PHYRE, GenThreader, FORTE and webPRC [37]. More recent multiple sequence alignment program BCL::Align includes also combined evaluation of structural similarities [38] (for applications, see, e.g., [39, 40]). The corresponding scoring function is a weighted sum of scores derived based on (i) the traditional PAM, and BLOSUM scoring matrices, (ii) position-specific scoring matrices by PSI-BLAST, (iii) secondary structure predicted by a variety of methods, (iv) chemical properties, and (v) gap penalties. Monte Carlo algorithm was then used to determine necessary optimized weights in cases of sequence alignment and fold recognition.

Input of 3D coordinates or their transformed representations was necessary for other structural studies predicting also functional interaction sites. The corresponding research yielded two servers with different 3D-BLAST programs enabling us to compare folds and fold families [41, 42] (for details see also Table 1). Alternative structural comparison substituting 3D relationships was performed when searching for the maximum contact map overlaps [43]. The extended contact map comparison appeared very early [44]. Contact map is in fact determined by the matrix of distances between individual amino acids, contact threshold and specification of contact types [45, 46]. This map can be visualized by CMView software [46]. Similarly to sequence motifs, conserved patterns of 3D peptide arrangements (CP-3D-A) were considered as an additional type of structure-function motifs. These structures were recorded by sequence independent 3D-templates and can be searched on Evolutionary Trace Annotation server [47, 48]. Frequent functionally important CP-3D-A occurred, for instance, in active sites of proteins containing porphyrin rings including members of cytochrome P450 superfamily [49]. Another recent interesting approach consisted in generalized motif search in large alphabet (cumulative occurrence of several alphabets in this case) inputs including simultaneously evaluation of DNA sequence, protein sequence and supersecondary structure motifs [50]. According to the authors, large alphabets are important in cases when structures share little similarity at primary level.

Similarly to multiple sequence alignment, various forms of multiple structural alignments (MSTA) have been generated. Older attempts at MSTA were based on secondary structural alignment [51–53]. On the other hand, recent MSTA approaches determined common spatial (3D) structures. These approaches include new strategies employing “molecular sieving” of protein structures, minimization of an energy function over the low-dimensional space of the relative rotations, and translations of the molecules, geometric hashing and contact-window-derived motif library [54–58].

In spite of the increasing possibility of database reevaluation, some structurally based programs predicting interacting sites became at least relatively autonomous. A large number of specific and relatively autonomous programs concerned the prediction of epitopes (for details see Section 3). Similarly, various possibilities of prediction of phosphorylation sites were frequently investigated (two examples in Table 1), since phosphorylation reactions represent an important signaling and regulatory network in cell biology and pathobiology. The interest followed also from the extended building of databases related to phosphorylation sites, which brought many interesting insights. For instance, in accordance with the linear motif atlas for phosphorylation-dependent signaling, tyrosine kinases mutated in cancer exhibited lower specificity than their non-oncogenic relatives [59]. Similarly, collection and motif-based prediction of phosphorylation sites in human virus proteins suggested a substantial role for human kinases in regulation/mediation of viral protein functions [60]. In contrast to programs predicting specific types of interacting sites, SeSAW represents an example of general online accessible program allowing prediction of possible functionally important structures [61] (see also Table 1). The corresponding balance between different combined structural evaluations concerned, among others, data present in position specific scoring matrix (PSSM), template-derived PSSM and template functional annotations.

## 3. Mimotope and Epitope Interactions

Epitopes are defined as the structures responsible for interaction of antigens with binding sites of antigen receptors. On the other hand, mimotopes belong to artificially prepared peptides, which interact with natural templates (most likely proteins), and thus mimic other peptides or organic compounds in their functionally important interactions. Mimotope development is based on synthetic peptide or phage display libraries, whereas natural development of specific cell clones is necessary for epitope recognition by specific antigen receptors. This means that both epitopes and mimotopes can sometimes considerably differ from the usual conserved structures mentioned above. In spite of the described difference, a unifying point between mimotopes and epitopes exists, because mimotopes were originally defined as peptides mimicking epitopes [72], forming thus only a subset of the later current mimotope repertoire. In addition to this historical linkage, rapidly developing (diverging) structures such as molecular mimicry enabling parasitic attack and adaptation of pathogenic viruses and bacteria, disordered regions of proteins, and protein loops appear to be good candidates for extended investigation of mimotope similarities (cf. [21, 73–75]) and mimotope based prediction (see below).

Mimotopes were originally derived in studies with a phage display library. This phage technology was discovered in the eighties [76]. The corresponding boom in the nineties then comprised novel random, partially randomized or gene-fragment-derived oligopeptide libraries able to functionally mimic epitopes, autoepitopes, short peptide ligands, protein kinase or proteinase substrates, as well as peptides mimicking organic substances such as biotin when interacting with steptavidin [77–85]. Mimotope similarities were frequently defined using sequence patterns, whereas additional types of nonsequence structural similarities were also described (see, e.g., [81, 83]). More recent biotechnological research of mimotopes yielded potential peptide drugs [86–89], peptide vaccines [90–92], and peptides suitable for specific (mostly nanoparticle mediated) drug delivery to tumor cells, brain, atherosclerotic plaques, and other therapeutically important sites of human or animal organisms [93–97]. In spite of this considerable progress, virtual interactomic tools are not still able to compare or predict organic drugs based on their effective spatial similarity with functionally active mimotopes.

Database registration and authentication of mimotopes have been performed for more than ten years [98–100]. Special programs comparing primary structures of epitopes were simultaneously developed (programs FINDMAP, and EPIMAP [101, 102]). Some of them employed also multiple-sequence alignment evaluation (program MIMOP [103]). Similarly, coexisting peptide databases were established to process the accumulated information. These databases (i) recognized sequence subsets classified after *in vitro* evolution of phage display libraries, (ii) offered many integrating programs, and (iii) made it possible to find all mimotope sets that have the 3D structure of a target-template complex (databases ASPD, RELIC and MimoDB [98, 99, 104, 105]). In addition, novel mimotope-assisted computer-aided epitope prediction was discovered. This prediction came from both the 3D structure of an interacting partner and sequences of similarly interacting mimotopes, and has also concerned some interacting partners different from specific antigen receptors and antigens (programs PepSurf, Pep-3D-Search, and MimoPro [106–108]). Based on this approach, improved specificity, and extended the repertoire of predicted epitopes or other interacting partners were achieved. Further progress in programming then resulted in accelerated computation in spite of more complicated, and precise strategies of data processing. In addition, pattern recognition algorithm was developed, which can effectively be employed to screen a mixture of antibodies, and define the breadth of epitopes recognized by polyserum directed against specific proteins [109]. This possibility appears to be interesting with respect to future vaccine design.

The recent status of epitope prediction is still far from resolved due to insufficient extent of the datasets, and still requires continuous improvement of database organization [110, 111]. In such state, mimotope-assisted epitope prediction mentioned above and combined approaches represent a certain improvement in the quality of epitope prediction. Online accessible combined approaches of B-cell epitope prediction mostly evaluate 3D structures together with solvent accessibility (programs CEP, ElliPro, PEPOP and 3D alternative of Epitopia [112–115]; Table 2). Similarly to many other combined approaches, combined restriction diminishes the number of false positivities but simultaneously can cause increased number of false negativities. For instance, the widely used requirement of solvent accessibility mentioned above would in fact eliminate at least part of conserved autoepitopes, which contain hydrophobic patterns (cf. [116–118]). To diminish losses following from employed combinations or effects of too strict (sure) thresholds, a metaserver was developed that sums up the results from six epitope-predicting servers [119]. In addition, some new types of online accessible prediction of linear epitopes appeared to complete the preceding results [120, 121].

An interesting input simplification has been achieved with a novel web server CBTOBE. This server uses learning of support vector machines based on physicochemical profiles, and makes it possible to predict conformation epitopes based on a sole sequence input [122]. A recent private alternative of CBTOBE was based on older evaluation of secondary structure and solvent accessibility (server COBEpro [123]) further complemented by evolutionary information and machine-learning-derived evaluation [124].

Combined predictions of epitopes presented to T-cell receptors have integrated class I MHC (major histocompatibility complex) peptide binding affinity, TAP transport efficiency and prediction of proteasome cleavage (programs EpiJen, NetCTL-1.2, FRED [125–128]). Nevertheless, independent simulations of peptide-binding affinity of various MHC molecules have been also proposed [129] as well as the corresponding neural network-based learning [130]. Both combined, and sole approaches then represent starting steps for further reevaluation with respect to T-cell receptor interactions, for example, using learning of support vector machines, and strict kernels based on 531 physicochemical properties (POPISK [131]). Important information about existing T-cell, and B-cell epitopes can be also obtained from the Immune Epitope Database (IEDB [132, 133]). Among others, EpitopeViewer of the 3D structural subcomponent of IEDB (IEDB-3D) allows the user to visualize, render and analyze the structure, and save structural and contact views as high-quality pictures for publication [133].

Since production of various vaccines, and detection kits with mononoclonal antibodies requires high efficiency of preparations, special searches for conserved epitopes have been developed for this purpose. Though the meaning of crucial term “conserved epitope” exceeds primary structure relationships, the repertoires of the corresponding structures appear to be sometimes considerably limited. In fact, structural or regulatory adaptations of pathogenic microogranisms and viruses cause less stability of immunologically important, and promising promiscuous (cytotoxic or helper) T-cell epitopes broadly cross-reacting with different MHC antigens, different frequencies of T-cell epitopes specific usually only for certain unique MHC molecule, as well as losses of immunogenicity or even absence of immune response. In addition to current evaluation, drug effects (cf. effects in docking studies described below) and accompanying structural variability (e.g., enzyme polymorphisms or special pathogenetical effects) have to be considered with respect to possible peptide epitope modifications, possible bias, or improvement of therapeutical design. It is a question whether conserved spatial structures following from multiple structural alignments mentioned above can be also interesting for prediction of conserved conformation of B-cell epitopes. The second part of Table 2 contains some web servers interesting with respect to prediction of conserved epitopes, whereas selected examples related to AIDS research follow.

The first more complex prediction of HIV epitopes was based on sequence conservativeness, secondary structure, solvent accessibility, hydrophilicity and flexibility [134]. The study pointed to an unfavorably frequent occurrence of changes in secondary structures predicted as antigenic. Certain progress in the research of conserved HIV-1 epitopes was achieved when assessing their possible interactions with MHC antigens. This comprised peptide prediction based on EpiMatrix score [135], construction of the peptide property model from a training dataset [136, 137] and combined T-cell epitope evaluation mentioned above [126, 127]. Lately, phylogenetic hidden Markov models allowed to predict HIV-1-related T-cell epitopes based on contiguous aa positions that evolve under immune pressure dependent among others on host HLA alleles [138]. In a recent paper, structure-function analysis based on a specifically devised mathematical model revealed that protection from neutralization (shielding of neutralization-sensitive domains) is enforced by intersubunit contact between the variable loops 1 and 2 (gp120 V1V2) of HIV-1, and domains of neighboring gp120 subunits in the trimer encompassing the V3 loop [139].

## 4. Protein Docking

Molecular docking is a method, which predicts the preferred reciprocal orientation of two molecules when they bound to each other to form a stable complex [147]. In case of simulated protein interactions, the authors currently speak about protein docking rather than about molecular docking, since molecular docking represents a term comprising also nucleic acid interactions with nonpeptide molecules. Formerly, molecular docking simulated “*lock-and-key*” type of protein interactions. Its original variants appeared in the eighties and reassumed interpretations of older molecular graphics [148–152]. These approaches were restricted by complementarity demands or simplified requirements for energy minimization. Lately, “*hand-in-glove*” analogy was found to be more appropriate for molecular docking than the “*lock-and-key*” one [153]. In addition to this conventional model, three new models have been recently developed for docking studies of protein-protein interactions [154], that is, (i) conformer selection model, using a novel ensemble docking algorithm, (ii) induced fit model, employing energy-gradient-based backbone minimization, and (iii) combined conformer selection/induced fit model. Physics-based molecular mechanics allowed the development of the force fields, which enabled the assessment of relative binding strength [155]. Alternative knowledge-based approaches were evolved to derive a statistical potential for interactions from a large database of protein-ligand complexes [156]. Affinity evaluation, statistical methods and combined procedures represent frequent alternatives of evaluation in recent protein docking approaches (Table 3). In addition to usual full 3D models simulating molecular complexes, contact docking was also proposed. This docking is based on contact map representations of molecules (cf. chapter 3). According to the authors contact docking appears feasible, and is able to complement other computational methods for the prediction of protein-protein interactions [157].

The number of molecules whose interactions can be scanned by current protein docking depends usually on the complexity of evaluated interactions and manner of simulation. For instance, interaction of 142 drugs inhibiting Poly-(ADP-ribose)-polymerase was tested in a large-scale virtual docking screening together with 300 000 added organic compounds with the aid of the program Lead Finder [158], whereas only 176 protein-protein interactions were analyzed by efficient program ZDOCK during extended simulation [159]. In addition to current output, some docking studies even verified the results of simulation using *in silico* experiments. For instance, aa involvement in enzyme interactions was proved with simulated mutation (program SYBYL 6.7 and Internal Coordinate Mechanics method [160, 161]) whereas other authors compared separate simulated effects of inhibitors and substrates (program BioDock [162]).

In addition to current molecular docking simulations (Table 3), certain docking studies analyzed modulation effects of additional molecules on the evaluated interaction [163–165]. Homology modeling followed by extensive molecular dynamics simulation was used to identify nonpeptidic small organic compounds (among 150 000 compounds) that bind to a human leukocyte antigen HLA-DR1301, and block the presentation of myelin basic protein peptide (aa positions 152–165) to T-cells. This peptide represents one of the epitopes critical for multiple sclerosis. *In silico* selection resulted in a set of 106 small molecules, two lead compounds were confirmed to specifically block IL-2 secretion by DR1301-restricted T-cells in a dose-dependent and reversible manner [163].

Pockets opening to protein surface represents potential sites for ligand binding or protein-protein interactions that were indeed identified in some cases [166–168]. Consequently, an algorithm BDOCK facilitating pocket-based prediction of protein binding site was proposed to improve protein docking [169], and moreover new algorithms predicting pockets are still proposed [170]. Many pockets were only transiently present on simulated surfaces of investigated proteins (e.g., Bcl-xl, interleukin 2, MDM2), and were all opened only 2.5 picosecond when using model intervals corresponding to ten nanosecond range [171].

Some docking studies comprised phylogenic aspects of protein interactions. It was observed that except for antibody-antigen complexes [172], the surface density of conserved residue positions at the interface regions of interacting protein surfaces is high. The corresponding combination of the residue conservation information with a widely used shape complementarity algorithm improved the ability of protein docking to predict the native structure of protein-protein complexes. Efficient comparative docking consists in selection based on conservation in terms of chain positions and primary structure in the first step, and the following high-throughput structure-based docking evaluation [173] (see also Table 3). Recent combined strategy including multiple sequence alignment and fold recognition analysis has been proposed to perform more precise docking, and predict also protein function [174].

Bioinspired algorithms were also applied to molecular docking simulations including neural networks, evolutionary computing and swarm intelligence [175]. Though neural networks participated in some older evaluations working in conventional programs of molecular docking [176–179], their independent scoring functions for docking have appeared lately [180, 181]. An extreme neural network approach required only sequence input to perform a docking-like procedure [182]. Predictions followed from a trained model that simulated binding or nonbinding stages.

## 5. Protein Interaction Networks

Protein interaction networks (PINs) are usually represented by graphs. Nodes of these graphs denote interacting molecules, whereas each edge linking two nodes indicates the corresponding interaction. The prevailing part of PIN is usually constituted by protein-protein interaction network (P-PIN). In addition to P-PIN, we can observe a record of protein interactions with (i) nonpeptidic hormones or mediators and drugs, (ii) processed, targeted, and functional complexes forming RNA, and (iii) recombining, hypermutating, repairing, replicating, twisting/untwisting, and transcribed DNA. These nonpeptidic compounds represent in fact inputs, outputs, interaction-stabilizing agents, or relay batons of P-PIN. In cases of DNA, and RNA, some papers appeared dealing with special protein-RNA, and protein-DNA interaction networks [205–208]. Protein-DNA networks moreover combined both the protein-centric and the DNA-centric points of view [205].

Like in other related networks, clusters are recognized in P-PIN. Two main types of P-PIN-related clusters can be distinguished, that is, protein complexes and functional modules [209, 210]. Protein complexes are groups of proteins that interact with each other at the same time and place, forming a single multimolecular machine (e.g., metabolic multienzyme complexes). Functional modules consist of proteins that participate in a particular cellular process while binding to each other at a different time and place (e.g., multiple signaling cascades are functional modules sometimes including also protein complexes). An important type of functional modules, that is, responsive functional modules (RFMs), includes protein interactions activated under specific conditions. These RFMs appear to be interesting with respect to prediction of potential biomarkers [211].

Much attention has been paid to the identification of small conserved subgraphs, particularly those occurring significantly often within the biological networks. These conserved subgraphs were referred to as network motifs or simply as motifs (similarly to primary structure motifs), and were considered as basic building blocks of complex networks including P-PIN [212, 213]. Ancestral pathways or functional modules including conserved interactions were derived when comparing P-PIN of phylogenetically distant species such as human, *Saccharomyces cerevisiae, Drosophila melanogaster* and *Caenorhabditis elegans*, and various bacterial species [214, 215]. Proteins necessary for proteasome function, transcription, RNA processing and translation were frequent in conserved subgraphs of compared *Eukaryota*, whereas DNA repair proteins prevailed in conserved prokaryotic subgraphs. Incorporation of literature-curated and evolutionarily conserved interactions also allowed to develop an interaction network for 54 proteins providing a tool for understanding Purkinje cell degeneration [216]. This result made it possible to find experimentally 770 mostly novel protein-protein interactions using a stringent yeast two-hybrid screen. 

Recent integrative approaches combined at least transcriptional data with P-PIN analyses, and at most five layers including phenotype association with single-nucleotide polymorphism, disease-tissue, drug-tissue and drug-gene relationships, in addition to the topical P-PIN record [217] (see also Table 4). Two-layer comparison of disease-related mRNA expression, and human P-PIN was for instance used together with hierarchical clustering of networks to elucidate human disease similarities. The results led to a hypothesis considering common usage of some drugs in the diseases, which exhibited close relationship with respect to given clustering [218]. More extended analysis of gene expression overlays with protein-protein interaction, transcriptional-regulatory and signaling networks identified distinct driver-networks for each of the three common clinical breast cancer subtypes, that is, oestrogen receptor positive, human epidermal growth factor receptor 2 positive and triple receptor-negative breast cancers [219]. Integrative online accessible tools reevaluating P-PIN relationships were developed to predict candidate genes critical for the occurrence of different diseases (cf. network-based disease gene prioritization; [220, 221]).

The majority of proteins reciprocally interact via one or two interactions. This number substantially increases to tens, hundreds and more, when considering multi-interactive proteins denoted in PIN as hubs [222]. In agreement with the sense of the term, hubs are the principal agents in the interaction network and affect its function and stability [223]. Hubs were enriched in kinase and adaptor proteins including those interacting with frequently disordered phosphorylated protein regions [224, 225]. Similarly, many pathogenetically important proteins belonged to hubs, for example, the widely known tumor suppressor p53, alpha-synuclein involved in Parkinson disease and small multifunctional core protein necessary for orchestration of viral progeny in *Flaviviridae* [226, 227]. In principle, two types of hubs were distinguished, that is, static “party hubs” and dynamic “date hubs.” Party hubs are markedly more phylogenetically conserved, and their expression is highly correlated with their interacting partners in contrast to less conserved date hubs more frequently containing disordered regions and participating in cell signaling [228–230]. Hubs with two or more domains are more likely to connect distinct functional modules than single-domain hubs [224]. In addition to studies of biochemically interesting hubs, docking procedures (mediated by program ClusPro [231]) were recently employed together with comparative modeling to construct P-PIN [232], whereas domain-domain, domain-motif and motif-motif interactions were distinguished in some other P-PIN [233].

Extended P-PIN research yielded many tools allowing P-PIN-based prediction of protein complex formation [234–237], identification of hubs and multifunctional proteins [238, 239], identification of functional modules [240–242], network-based disease gene prioritization [217, 221, 243], as well as network building [244, 245], cross-species querying [246] or comparison [234, 237]. Some of these approaches as well as several examples of P-PIN-related databases are described in Table 4. The instrumental progress in PIN research also led to an increased number of the corresponding methodological reviews, for example, [247–250].

## 6. Accuracy as an Important Parameter

A detailed evaluation of accuracy of all the above approaches would require a separate review. The obstacles consist first of all in presence of complementary information in articles inaccessible for biologists and on web pages. Such state complicates mining of accuracy data first of all in the case, when server-related papers represent only the final step of author's efforts. Accuracy evaluation or even the corresponding references are also rarely present in the papers concerning new online databases. In addition, some authors also use other criterions related to accuracy to evaluate the corresponding performance value, whereas only some of such evaluations are widely known as valid accuracy substitution, for example, AUC (area under the ROC curve) discussed below.

In spite of the obstacles, we can mention several examples of high accuracy concerning the above approaches. Excellent accuracy values higher then 0.90 were found in the cases of Conserved Domain Database (or its RPS BLAST server; [257]; Table 1) and older 3D-BLAST variant under a broad range of conditions (Table 1), whereas similarly interesting AUC values (higher than 0.90) were mostly found also in the cases of recent 3D BLAST variant (Table 1), and the selected strategy of SVM- and machine-learning-based epitope prediction known as CBTOBE (chapter 3; [122]). The same accuracy levels were rarely achieved in certain subsets when employing ADAN, KinasePhos 2.0 (Table 1), ElliPro, RANKPEP (Table 2), SVM and machine-learning-based epitope prediction improved by evolutionary information BEOracle (chapter 3; [124]) and docking tool HADDOCK (chapter 4; [178]). Similarly, restricted subset-related AUC values of 0.90 and 0.93 indicated a good predicting ability when using docking-like Partner-aware prediction (chapter 4; [182]), and combined evaluation based on Glide X and molecular mechanics (Table 3) to simulate interactions of 25 antibodies and P-glycoprotein, respectively. Frequent subset accuracies of higher values than the gold standard value of 0.80 were observed during runs of programs Phos3D, KinasePhos 2.0 (Table 1), ElliPro (Table 2) and CBTOBE mentioned above (chapter 3; [122]), and two docking tools Glide XP and Gold (Table 2; [258]). Comparable AUC values then concerned RANKPEP (Table 2), BEOracle mentioned above (chapter 3; [124]) and network-based disease gene prioritization called DADA (chapter 5; [236]). The low occurrence of P-PIN in our overview of accuracy evaluation followed from the fact that the performance of P-PIN was alternatively evaluated with the aid of precision or recall. An interesting approach—Lead Finder—was developed to improve the accuracy of protein-ligand docking, binding energy estimation and virtual screening. High enrichment factors were obtained for almost all of the targets and seven different docking programs resulting in an excellent average AUC value of 0.92 [258].

## 7. Tendencies, Improvements, and Interplays

In summary, virtual interactomics represents a highly sophisticated, and relatively autonomous subarea of both interactomics and bioinformatics. Its development can be characterized by several independent and integrative tendencies. The independent tendencies include (i) more rapid and precise molecular modeling of protein complexes (including quantum mechanic methods and rapid pharmacological screening based on docking), (ii) more complex evaluation of structural similarities (e.g., large alphabets and combined prediction of functional sites), (iii) extensive assembly of large-scale PIN and assembly of PIN or PIN subgraphs with dynamical properties, and (iv) restriction of conserved PIN subgraphs and clinically interesting responsive functional modules. The integrative tendencies then comprise (i) establishment of structurally functionally, and purposefully oriented databases and associated data mining servers, (ii) questions concerning phylogenic relationships between structural differences in PIN subgraphs, and the differences in protein structures of subgraph forming molecules, (iii) PIN prediction and revision/reevaluation of empirically proposed PIN using comparative molecular modeling, docking and bio-inspired algorithms, (iv) comparative differential multilayer network approaches including clinical treatment cases, and (v) attempts to construct global networks within a single cell, and hopefully, within the system of different reciprocally communicating cell types (e.g., immune system). Since interactomics simplifies reactomic simulations, it can also markedly and rapidly complete reactomic results, for example, in prediction of enzyme-inhibitor effects and specificity of protein kinase interactions mentioned above. In the end, we also dealt here with accuracy, which represents an important parameter of tool choice together with reasonable tool accessibility, and possibilities to test several alternatives before prediction, simulation and network analysis or to use two or several independent methods. In conclusion, many interesting and important interplays occur inside the virtual interactomic area as well as in its boundary lines and performance evaluation.

## Figures and Tables

**Table 1:** Some more recent online accessible bioinformatic tools.

Programs	Purpose	Input	Evaluation tools and procedures	Compared structures	Internet accessibility	References
Program tools						

3D-BLAST (two methods)	^ ∗^Identification of 23 states of the structural alphabet	PDB ACF	^ ∗^SASM	^ ∗^structural alphabet sequence databases	http://3d-blast.life.nctu.edu.tw/	[41, 42]
	^ ∗^Comparison of protein folds using spherical polar Fourier basis functions.		^ ∗^Carbo-like similarity score	^ ∗^SPF shape density rep.	http://threedblast.loria.fr/	
DASMIweb	Online integration, analysis and assessment of distributed protein interaction data and predictions (interactomic mining)	some protein identifiers	literature curation, prediction, 3D analysis	records of interactions	http://www.dasmiweb.de	[17]
FFAS03	Server accepts a user supplied protein sequence and automatically generates a profile, which is then compared with several sets of sequence profiles of proteins databases	QS	PPA	DS	http://ffas.godziklab.org/	[36, 37]
KinasePhos 2.0	Prediction of kinase-specific phosphorylation sites based on the site sequences from databases	QS	k-fold + Jackknife cross-validations	phosphorylation sites from Phospho.ELM Swiss-Prot	http://KinasePhos2.mbc.nctu.edu.tw/	[62]
Phos3D	Method of phosphorylation-site prediction based on 3D structural information associated with 530 phys-chem-pr	QS (+3D context)	SVM, spatial amino acid propensities	PDB coordinates	http://phos3d.mpimp-golm.mpg.de/	[63]
SeSAW	Identification of functionally or evolutionarily conserved motifs based on balancing between sequence and structural similarities	^ ∗^PDB QF, ID; ^∗^PDB QF, ID, IDt	*P* value SeSAW score	conserved domains templates	http://sysimm.ifrec.osaka-u.ac.jp/SeSAW/	[61]

Databases						

ADAN	Prediction of protein-protein interactions of different modular protein domains mediated by linear motifs	PDB ACF	PSSM_PI	integrated DaSt	http://adan-embl.ibmc.umh.es/	[64]
CDD (RPS BLAST)	Conserved domain relationships, domain location of frequent binding sites	QS	model MSA derived PSSM	Conserved domains (PSSM)	http://www.ncbi.nlm.nih.gov/Structure/cdd/cdd.shtml http://www.ncbi.nlm.nih.gov/BLAST	[65–67]
GWIDD	Docking database; integrated resource for studies of protein-protein interactions on the genome scale	search interface	docking techniques	DaSt	http://gwidd.bioinformatics.ku.edu	[68]
MegaMotifBase (structural database)	3D motif orientation, intermotif distances, solvent accessibility, secondary structure content, hydrogen bonding, residual packaging, familiar/superfamiliar/none motif relationship	motif or sequence pattern	complex evaluation including projection	DS + DaSt	http://caps.ncbs.res.in/MegaMotifbase/index.html	[69]
PTGL	Database for secondary structure-based protein topologies; visualization of topology diagrams and 3D structures	PDB QF	ULN4D_graph theory	DS + DaSt	http://ptgl.uni-frankfurt.de/	[70]
RsiteDB	Database of protein binding pockets which interact with single strand RNA	PDB code, UPS	3D arrangement of phys-chem-pr	3D-CBP	http://bioinfo3d.cs.tau.ac.il/RsiteDB/	[71]

^∗^Independent alternatives; 3D-CBP: 3D consensus binding patterns important for protein-nucleotide recognition; DS: database sequences; DSA: double sequence alignment; DaSt: database structures; CS: list of compared sequences; ID, IDt: chain or template ID, respectively; MSA: multiple sequence alignment; QS: query sequence(s) (instead of QS, clonal names or gi numbers can be used in majority of given approaches); phys-chem-pr: physicochemical properties; PDB: Protein data bank; PDB QF: PDB-formatted query file; PDB ACF: PDB-derived atom coordinate file; PPA: profile-profile alignment; PSSM: position-specific scoring matrices; PSSM_PI: PSSM for protein-protein interactions calculated by FoldX; rep.: representations; SASM: structural alphabet substitution matrix; SPF: spherical polar Fourier; SVM: support vector machines; ULN4D_graph theory: unique linear notations of four descriptions for protein structures on different abstraction levels based on graph theory; UPS: unbound protein structure.

**Table 2:** Epitope prediction or reevaluation on accessible servers.

Programs	Purpose	Tools or procedures of evaluation	Input	Output	Internet accessibility	References
Epitope prediction						

MimoDB 2.0	Mimotope database	MySQL relational database	according to menu	Structures visualization, alignments, and so forth	http://immunet.cn/mimodb/	[105]
MimoPro	Maps a group of mimotopes back to a source antigen so as to locate the interacting epitope on the antigen	Branch and bound optimization (analysis of overlapping patches on the surface of a protein)	PDB identifier mimotope sequences	Score + 3D location in antigen	http://informatics.nenu.edu.cn/MimoPro/	[108]
ViPR	Virus pathogen database	Integration of various resources	according to menu	Structures, annotations, and so forth	http://www.viprbrc.org/	[140]
MetaMHC	Prediction of MHC binding epitopes	meta-approach	QS	Four metapredictor scores including MetaSVMp score	http://www.biokdd.fudan.edu.cn/Service/MetaMHC.html	[141]
Epitopia	Prediction of B-cell epitopes	^ ∗^MLA ^∗^MLA + 3D + SA	^ ∗^QS ^∗^PDB ACF	Immunogenicity score + probability score + color scale record on QS	http://epitopia.tau.ac.il	[115]
ElliPro	New structure-based tool for the prediction of antibody epitopes	3D + SA + flx + antigenicity	^ ∗^QS ^∗^PDB ACF	Score + visualized epitope 3D structure and 3D location	http://tools.immuneepitope.org/tools/ElliPro	[114]
MHCPred	Prediction of class II mouse MHC peptide binding affinity	ISC-PLS, SYBYL software package	QS	Binding affinity (pIC50)	http://www.jenner.ac.uk/MHCPred	[129]

Stable epitopes and vaccines						

BayesB	SVM-based prediction of linear B-cell epitopes using Bayes Feature Extraction	Residue conservation + position-specific residue propensities	QS	Residue epitope propensity score	http://www.immunopred.org/bayesb/index.html	[120]
OptiTope	Selection of optimal peptides for epitope-based vaccines	Multistep parallel evaluation	MSA of Ag list of ES table: ES, ES related MHC	Fraction of overall immunogenicity covered MHC	http://www.epitoolkit.org/optitope	[142]
PVS	PVS returns a variability-masked sequence, which can be submitted to the RANKPEP server to predict conserved T-cell epitopes.	3D visualization of MSA derived sequence variability “per site”	PDB ACF	3D map of variability fragments with no variable residues and their 3D location in antigen	http://imed.med.ucm.es/PVS/	[143]
PEPVAC	Prediction of MHC I; server can also identify conserved and promiscuous MHC I ligands	PSSM, distance matrix, phylogenic clustering algorithm	Genome, HLA-supertypes	Selected peptide sequences score	http://immunax.dfci.harvard.edu/PEPVAC/	[144]
RANKPEP	Prediction of MHC I and MHC II ligands	Profile comparison	Genome, HLA-supertypes, QS	Selected peptide sequences score	http://www.mifoundation.org/Tools/rankpep.html	[145, 146]

^∗^Independent alternatives; Ag: antigen; BFE: Bayes Feature Extraction; flx: flexibility; ES: epitope sequences; HLA: human leukocyte antigens (human MHC); ISC-PLS: iterative self-consistent partial-least-squares based additive method; MHC: alleles of major histocompatibility antigens; MLA: machine learning algorithm; MSA: multiple sequence alignment; PDB: protein data bank; PDB ACF: PDB-derived atom coordinate file; QS: query sequence(s); SA: solvent accessibility; SVM: support vector machines.

**Table 3:** Examples of protein docking.

Approach/combination	Investigated molecules	Predicted/investigated partners	Evaluation tools and results	Employed internet addresses	References
Protein-ligand					

SwissDock	protein	small molecule (ligand)	FullFitness + RMSD	http://www.swissdock.ch	[183]
Glide (version 5.6)	P-glycoprotein	24 P-gp binders + 102 endogenous molecules	Glide XP score + MM-GB/SA rescoring function	http://pubchem.ncbi.nlm.nih.gov/	[184]
VSDocker (AutoGrid4, AutoDock4, etc.)	protein	small molecule (example with 86 775 ligands)	Δ*G*_bind_	http://www.bio.nnov.ru/projects/vsdocker2/	[185]
Opal (Autodock tools)	protein	small molecule (ligand)	Δ*G*_bind_	http://kryptonite.ucsd.edu/opal2/dashboard?command=serviceList	[186]
FlexX module of SYBYL 7.0 and FMO	neuraminidase (from N1 subtype of influenza virus)	carboxyhexenyl derivatives or analogues	FlexX energy scores	http://www.rcsb.org/pdb http://grid.ccnu.edu.cn/	[187]
TarFisDock	protein	drugs	interaction energy	http://www.dddc.ac.cn/tarfisdock/	[188]
KinDOCK	ATP binding sites of PK	focused ligand library	theoretical affinity	http://abcis.cbs.cnrs.fr/kindock	[189]
AutoDock + Gold	Cdc25 dual specificity phosphatases	library of sulfonylated aminothiazoles as inhibitors	Δ*G*_bind_ + overall Gold fitness function	none	[190]
Molsoft module + ACD database	thyroid hormone receptor	250 000 compounds	EQS score	none	[191]

Protein-protein					

ZDOCK	protein	flexible/shape complementary protein	combined evaluation using Fast Fourier Transform	http://zlab.umassmed.edu/zdock/ http://www.fftw.org	[192] [159, 193]
KBDOCK	protein	protein with hetero-binding site	combined knowledge-based approach	http://kbdock.loria.fr/	[194]
RosettaDock	protein	protein	CAPRI CS	http://rosettadock.graylab.jhu.edu	[195]
ClusPro	protein	protein	combined evaluation	http://cluspro.bu.edu/login.php	[196, 197]

Protein-nucleic acid					

DARS-RNP QUASI-RNP	protein	RNA	knowledge-based potentials for scoring protein-RNA models	http://iimcb.genesilico.pl/RNP/	[198]
protein-DNA docking approaches	transcription factor/protein	DNA	RMSD + knowledge based energy	http://pqs.ebi.ac.uk/ http://sparks.informatics.iupui.edu/czhang/ddna2.html	[199] [200]

Antigen receptors					

DS-QM based peptide binding prediction	HLA-DP2	HLA-DP2 interacting potential TCR ligands	CS following from two different DS-QM	none	[201]
SnugDock	antibody	antigen	paratope based structural optimization + CLEP	http://www.rosettacommons.org/	[202]
pDOCK	MHC I and II	potential TCR ligands	RMSD	http://biolinfo.org/mpid-t2	[203]
MHCsim	MHC I and II	potential TCR ligands	molecular dynamic simulation	http://igrid-ext.cryst.bbk.ac.uk/MHCsim/	[204]

ACD: available chemicals directory (database); CAPRI: critical assessment of predicted interactions; CLEP: combined lowest energy prediction; CS: combined scoring; DS-QM: docking score-based quantitative matrices; EQS score: score combining grid energy, electrostatic and entropy terms; FMO: fragment molecular orbitals; HLA-DP: a subset of human MHC II; Δ*G*_bind_: free energy of binding; PK: protein kinases; MM-GB/SA molecular mechanics scoring function with generalized Born implicit solvent effects; P-gp: P-glycoprotein; RMSD: root mean square displacement statistics; TCR: T-cell receptor.

**Table 4:** Online accessible tools for PPI network assembly, reevaluation, and comparison.

	Purpose	Input	Evaluation results	Network content	Internet accessibility	References
Program tools						

PINTA	Resource for the prioritization of disease related candidate genes based on the differential expression of their neighborhood in a genome-wide protein-protein interaction network.	Files with disease-specific expression data	Internal scores, *P* values, Bonferroni–Holm *P*-values	menu: human, mouse, rat, worm (*C. elegans*) and yeast P-PIN	http://www.esat.kuleuven.be/pinta/	[221]
PCFamily	Finds homologous structure complexes of the query using BLASTP to search the structural template database	QS or QS set in FASTA format	*Z*-value agreement ratio	941 protein complexes	http://pcfamily.life.nctu.edu.tw	[236]
BisoGenet	Build and visualize biological networks in a fast and user-friendly manner including P-PIN	List of identifiers	Node degrees, cluster coefficients	5365 human genes	http://bio.cigb.edu.cu/bisogenet-cytoscape/	[245]
DynaMod	Identifies significant functional modules reflecting the change of modularity and differential expressions that are correlated with gene expression profiles under different conditions	Genome-wide expression profile	*Z*-score, Bonferroni or FDR *P*-values	In frame of GSEA	http://biocondor6.kaist.ac.kr/dynamod/	[241]
TORQUE	Cross species querying allows users to run topology-free queries on predefined or user-provided target networks resulting in subnetwork of the target network most similar to query subset	Seq of compared sets, query list, target P-PIN	Thickness of the edge	5430 proteins, 39 936 interactions	http://www.cs.tau.ac.il/~bnet/torque.html	[246]
NetworkBLAST	Identifies protein complexes within and across species, forming html page with links to schemes of interactions within complexes and additional graph data files	Files with ^∗^PPI, BLPE ^∗^PPI	likelihood based density score	species related P-PIN ^∗^two species ^∗^single species	http://www.cs.tau.ac.il/~roded/networkblast.htm	[234]

Databases						

STRING	A database of functional interaction networks of proteins, globally integrated and scored	Search based on gene annotations	probabilistic confidence score	>1100 completely sequenced organisms	http://string-db.org	[251]
ChemProt	Disease chemical biology database including 7 × 10^5^ chemicals and two millions chemical-protein interactions; indicates a possible formation of disease-associated protein complexes	Compound and protein identifiers	compilation of multiple chemical-protein annotations	30 578 proteins 2227 disease-related proteins, 428 429 PPI	http://www.cbs.dtu.dk/services/ChemProt/	[252]
NetAge	Database and network analysis tools for biogerontological research (integrity and functionality of P-PIN is under a tight epigenetic control)	Tools for searching and browsing	experimental evidence about interactions	miRNA-regulated P-PIN in age-related diseases	http://www.netage-project.org	[253]
BioGRID	All interactions in BioGRID are available through the Osprey visualization system, which can be used to query network organization in a user-defined fashion	Intuitive graphical interface	experimental evidence about interactions	Over 198 000 genetic + protein interactions from six species	http://www.thebiogrid.org	[254]
EHCO	Encyclopedia of Hepatocellular Carcinoma genes Online collect, organize and compare unsorted HCC-related studies	CMS	NL processing and softbots	97 proteins, 47 highly interactive, 18 hubs	http://ehco.iis.sinica.edu.tw	[255]
IntNetDB v1.0	A database providing automatic prediction and visualization of PPI network among genes of interest; analysis includes domain-domain interactions, known gene contexts, crossvalidation, and so forth	list of query gene identifiers	likelihood ratio following from Bayesian analysis	concerns 27 species contains GSP and GSN from HPRD	http://hanlab.genetics.ac.cn/IntNetDB.htm	[256]

^∗^Independent alternatives; BLPE: BLASTP *E* values between pairs of proteins from each of the compared species; CMS: content management system; GSEA: gene set enrichment analyses; GSP, GSN: gold standard positive and a gold standard negative dataset of HPRD, respectively; HCC: hepatocellular carcinoma; HPRD: human protein reference database; NL: natural language; PE: probabilistic evaluation; PPI: protein-protein interactions; QS: query sequences; seq: sequences.

## Abbreviations

3D: Three-dimensional
aa: Amino acid residues in protein sequence
PIN: Protein interaction network(s)
P-PIN: Protein-protein interaction network(s).
